# Amino-Modified Graphene Oxide from Kish Graphite for Enhancing Corrosion Resistance of Waterborne Epoxy Coatings

**DOI:** 10.3390/ma17051220

**Published:** 2024-03-06

**Authors:** Shengle Hao, Siming Wan, Shiyu Hou, Bowen Yuan, Chenhui Luan, Ding Nan, Gen Huang, Deping Xu, Zheng-Hong Huang

**Affiliations:** 1 School of Chemical & Environmental Engineering, China University of Mining & Technology (Beijing), Beijing 100083, China; hsl950219@163.com (S.H.); wansm1997@outlook.com (S.W.); luanchenhui2021@163.com (C.L.); huanggencumtb@163.com (G.H.); 2 Key Laboratory of Advanced Materials (MOE), School of Materials Science and Engineering, Tsinghua University, Beijing 100084, China; housy@tsinghua.edu.cn; 3 Inner Mongolia Key Laboratory of Graphite and Graphene for Energy Storage and Coating, School of Materials Science and Engineering, Inner Mongolia University of Technology, Hohhot 010051, China; 18340363303@163.com (B.Y.); nan1980732@163.com (D.N.); 4 College of Chemistry and Chemical Engineering, Inner Mongolia University, Hohhot 010021, China

**Keywords:** kish graphite, modified graphene oxide, 2-aminomalonamide, waterborne epoxy coatings, anti-corrosion

## Abstract

Waterborne epoxy (WEP) coatings with enhanced corrosion resistance were prepared using graphene oxide (GO) that was obtained from kish graphite, and amino-functionalized graphene oxide (AGO) was modified by 2-aminomalonamide. The structural characteristics of the GO and AGO were analyzed using X-ray diffraction (XRD), Raman spectroscopy, Fourier transform infrared spectroscopy (FT-IR), scanning electron microscopy (SEM), and transmission electron microscopy (TEM). And the anti-corrosive performance of waterborne epoxy-cased composite coatings with different addition amounts of AGO was investigated using electrochemical measurements, pull-off adhesion tests, and salt spray tests. The results indicate that AGO15/WEP with 0.15 wt.% of AGO has the best anti-corrosive performance, and the lowest frequency impedance modulus increased from 1.03 × 10^8^ to 1.63 × 10^10^ ohm·cm^−2^ compared to that of WEP. Furthermore, AGO15/WEP also demonstrates the minimal corrosion products or bubbles in the salt spray test for 200 h, affirming its exceptional long-term corrosion protection capability.

## 1. Introduction

To meet the challenge of metal corrosion and the goal of low volatile organic compound (VOC) emissions [1,2], it is an urgent need to develop environmentally friendly anti-corrosion measures that can offer superior protection in heavy-duty anticorrosive coatings. Waterborne epoxy (WEP) coatings are distinguished by their superior adhesion, minimal curing shrinkage, impressive mechanical properties, and negligible VOC emissions [3,4]. However, during the curing process of WEP, micro-porosity and micro-cracking can be formed because of the volatilization of solvent water, thus significantly affecting the barrier properties and reducing the life of the coatings when compared to organic epoxy coatings [5]. Previous research has demonstrated that incorporating 2D nanomaterials into water-based resins leads to a substantial improvement in the passive barrier properties [6,7]. Two-dimensional nanomaterials can fill the defects created during water evaporation, effectively blocking the contact between oxygen and water vapor in the environment and WEP. However, achieving compatibility between nanofillers and water-based resins poses a formidable challenge [8,9]. Due to the abundant oxygen-containing functional groups at the surface and edges of graphene oxide (GO) [10,11], GO exhibits excellent dispersion in water-based resin systems, which has great potential in the application of water-based anti-corrosion coatings.

Some studies have shown that the inclusion of GO in water-based epoxy resins can enhance the barrier properties of coatings. Jiang et al. [12] studied the impact of different aspect ratios of GO on the corrosion protection performance of GO/epoxy coatings; it was found that GO sheets with higher aspect ratios provide a more convoluted path for the corrosive medium to result in an improved corrosion protection performance. Due to the strong chemical bonds between epoxy and amino groups, researchers have utilized functional modifications of amino groups to enhance the performance of GO. Table 1 lists the preparation method and application of amino-modified GO. Nan et al. [13] successfully synthesized an amino-functionalized graphene oxide (DGO) and observed a remarkable enhancement in the corrosion resistance of epoxy coatings with the incorporation of DGO. This improvement can be attributed to the enhanced dispersion of GO within the coating matrix and the enhancement of its barrier properties. However, in light of the growing industrial demand for graphite (graphene), natural graphite is emerging as a pivotal strategic resource, witnessing a steady rise in prices [14,15]. So, it is necessary to identify alternative graphite-based materials that are readily accessible and cost-effective raw materials.

Kish graphite, a crystalline form of graphite formed by the precipitation of carbon from molten iron during cooling, is commonly found in the dust and slag of iron-making and converter steelmaking plants [20]. A substantial amount of kish graphite is considered solid waste in the iron and steel industry and has not been effectively utilized, resulting in significant resource wastage, environmental pollution, and potential security risks [21,22]. The presence of metal oxide impurities (such as Fe_2_O_3_, CaO, etc.) and structural imperfections often accompany the production of kish graphite, which hinders its large-scale application [23]. Currently, numerous research teams are engaged in the exploration of recycling and utilization methods for kish graphite. Li et al. [24] presented a comprehensive approach for the recycling of kish graphite, which possessed a high level of graphitization. Wang et al. [25] employed a comprehensive separation technique to concentrate and purify kish flake graphite (KFG). The KFG exhibited a satisfactory recovery rate of 57.34%. Additionally, KFG showcased a well-ordered-layer structure of graphite, resulting in an exceptional electrochemical performance. There have been studies on the preparation of exfoliated graphite by KFG and its application in the treatment of offshore oil spills [24]. However, there have been limited studies on the application of GO prepared by KFG for enhancing the corrosion resistance of WEP. Using KFG as a raw material to prepare GO will significantly reduce the cost of coatings, and the defects of KFG are also conducive to the modification of surface functional groups.

In this study, to improve the anti-corrosion properties of water-based epoxy resins to WEP, GO from industrial byproduct KFG and amino-functionalized GO (AGO) was prepared by 2-aminomalonamide. The AGO was employed as a functional filler in WEP to enhance the anti-corrosive performance. Furthermore, we examined the optimum incorporation of AGO and the corrosion protection performance of composite coatings. KFG used as a raw material to prepare GO can overcome the restriction of the high cost of natural graphite, which has a market application prospect in corrosion protection.

## 2. Experimental Section

### 2.1. Raw Materials

Concentrated sulfuric acid (98%, H_2_SO_4_), hydrochloric acid (36–38%, HCl), hydrogen peroxide (30%, H_2_O_2_), sodium nitrate (≥99.9%, NaNO_3_), and potassium permanganate (≥99%, KMnO_4_) were purchased from Tongguang Fine Chemical Co., Ltd. (Beijing, China). 2-Aminomalonamide (≥98%) was purchased from Shanghai Macklin Biochemical Co., Ltd. (Shanghai, China). Waterborne epoxy resin (MU-618, solid content was 48–52%) and waterborne epoxy curing agent (CU-600, solid content was 61–65%) were purchased from Shanghai Runtan New Material Technology Co., Ltd. (Shanghai, China). All reagents were used as received without further purification.

Q235 steel was purchased from Jixing Stainless Steels Factory (Beijing, Chian) and the chemical composition is listed in Table 2. They were polished in turn by SiC sandpaper with 400, 800, and 1200 mesh, then washed with acetone and dried at 60 °C. Kish flake graphite was supplied from the Qingdao Steel Mill (Qingdao, China), and the pristine kish graphite was subjected to a purification process involving water washing dust removal, magnetic separation, and acid leaching. The proximate analysis of purified kish graphite and chemical composition analysis of the ash are listed in Table 3.

### 2.2. Synthesis of GO and AGO

GO was synthesized by the modified Hummers method. Firstly, the purified kish graphite (0.5 g) was combined with H_2_SO_4_ (20 mL) and NaNO_3_ (0.25 g), with constant stirring for 2 h at the temperature below 15 °C. An amount of 3 g of KMnO_4_ was slowly added and further stirred at 35 °C for 0.5 h. Subsuquently, 40 mL of deionized (DI) water was slowly added into the solution and stirred for 15 min. Finally, 7.5 mL of H_2_O_2_ was added slowly until a bright yellow suspension appeared. The residual acids were removed using 5% HCl and DI water several times. GO powders were obtained after filtration and freeze drying.

**Figure 1:**
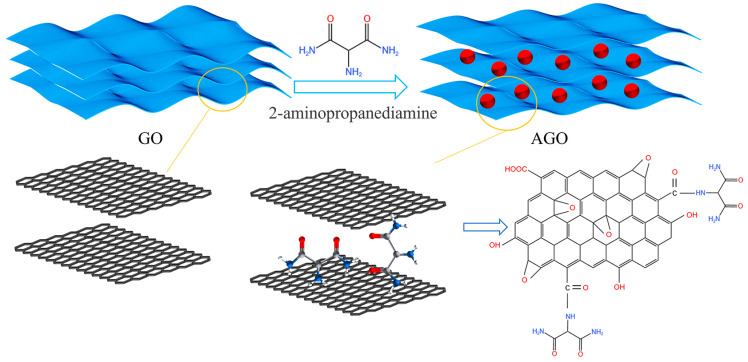
The mechanism diagram of AGO synthesis.

Figure 1 shows the mechanism diagram of the AGO synthesis. An amount of 0.2 g of GO and 20 mg of 2-aminomalonamide were dispersed in 50 mL of DI water, then ultrasonicated in an ice bath for 30 min. Next, the 2-aminomalonamide and GO solutions were mixed and stirred at 55 °C, 65 °C, 75 °C, and 85 °C for 1 h [26]. The final products were obtained by freeze drying, which were denoted as AGO55, AGO65, AGO75, and AGO85 to specify the respective reaction temperatures.

### 2.3. Preparation of GO/WEP and AGO/WEP Composite Coatings

AGO/WEP composite coatings were prepared by incorporating AGO65 (the optimum modification temperature) at mass fractions of 0.05%, 0.15%, and 0.3% relative to the solid weight of the resin and curing agent. Firstly, AGO65 was dispersed in DI water at the specified mass fractions and sonicated for 10 min to disperse it well. The evenly distributed mixture was combined with MU-618. Then, CU-600 with a stoichiometric amount was added to the mixture, maintaining a resin/curing agent weight ratio of 2:1. Finally, the prepared coatings were applied onto the pre-treated Q235 steel using a wire bar (100 μm) and cured at 60 °C for 24 h. The thickness of the coatings was controlled within the range of 40 ± 5 μm. The composite coatings were named AGO05/WEP, AGO15/WEP and AGO30/WEP by incorporating 0.05%, 0.15%, and 0.3% of AGO65, respectively. In addition, GO/WEP composite coatings were prepared with 0.15% GO, and the coating without AGO65 was named WEP.

### 2.4. Characterizations

X-ray diffraction (XRD, Bruker D8 Advance, Karlsruhe, Germany) was used to determine the crystal structure of the GO and AGO. Raman spectra were obtained with a Raman spectrometer (HORIBA, LabRAM HR Evolution, Tokyo, Japan) covering 500 to 4000 cm^−1^. Fourier transform infrared (FT-IR, Bruker VERTEX 70 V, Ettlingen, Germany) spectroscopy of the GO and AGO was conducted with a resolution of 4 cm^−1^ in the range of 4000–500 cm^−1^. The morphology and microstructure of the materials were observed using scanning electron microscopy (SEM, ZEISS GEMINISEM 500, Munich, Germany) and transmission electron microscopy (TEM, JEOL JEM-2100F, Tokyo, Japan). And the element mapping was also performed by EDS (X-MAX^20^, OXFORD INSTRUMENTS, Oxford, UK).

### 2.5. Electrochemical and Salt Spray Tests

Electrochemical impedance spectroscopy (EIS) and potentiodynamic polarization tests in a 3.5 wt.% NaCl aqueous solution that simulated a seawater environment were conducted to investigate the electrochemical properties of the composite coatings. These tests were performed on an electrochemical workstation (CHI-760E) equipped with a three-electrode configuration. The steel substrate coated with coatings (1 cm^2^) was the working electrode. Saturated calomel (SCE) was the reference electrode. EIS tests were conducted over a frequency range of 100 kHz to 0.01 Hz with an amplitude of 20 mV. Potentiodynamic polarization curves were plotted from an open circuit voltage (OCV) potential of −250–250 mV with a sweep rate of 1 mV/s. Electrochemical software (CHI-760E Version 21.02) was utilized to analyze the corrosion current density (*i_corr_*) and polarization resistance (R_p_). Additionally, the following equation was used to calculate the corrosion rate (*v_corr_*):

(1) $$v_{corr}=87.600\times \frac{E_{w}i_{corr}}{n\rho F}$$

where *E_w_* represents the formula weight of carbon steel (55.85 g/mol), *i_corr_* represents the corrosion density, *n* represents the chemical valence of ferric ion, *ρ* represents the density of carbon steel (7.86 g/cm^3^), and *F* represents the Faraday constant [27].

Salt spray tests were conducted on a Salt Spray Test Chamber for 200 h (YWX-015, Changzhou Guoli Test Equipment Co., Ltd. Changzhou, China) according to the standard ASTM B117-09 [28]. The saline concentration used in the test was 5 mg/mL, the PH value of the solution was between 6.5 and 7.2, and the test temperature was 35 °C.

## 3. Results and Discussion

### 3.1. Characterization of AGO and AGO/WEP Composite Coatings

**Figure 2:**
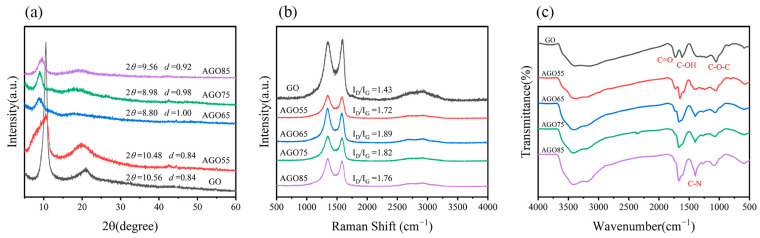
XRD (**a**), Raman (**b**), and FT-IR spectra (**c**) of GO, AGO55, AGO65, AGO75, and AGO85.

XRD spectra were used to analyze the chemical structures to demonstrate the modification effect, as shown in Figure 2a. The characteristic peak of GO (001) was determined according to the Bragg equation:

(2) 2dsinθ=λ

where *d* is the interlayer spacing of GO and AGO; *θ* is the grazing angle; and *λ* is the X-ray wavelength. A sharp diffraction peak appeared at 2*θ* = 10.56° in the GO sample, indicating an interlayer spacing of 0.84 nm. After the loading of 2-aminomalonamide, the diffraction peaks of AGO55, AGO65, AGO75, and AGO85 shifted to 10.48°, 8.80°, 8.98°, and 9.56°, with an interlayer spacing of 0.84, 1.00, 0.98, and 0.92 nm, respectively. The modified AGO samples successfully grafted the amino group and exhibited a larger interlayer distance, which was attributed to the reaction between GO and 2-aminomalonamide and the resulting intercalation between the GO sheets.

As shown in Figure 2b, Raman spectroscopy was used to characterize the disorder and defect structures of GO and AGO. Both GO and AGO exhibited a prominent D-band (1345 cm^−1^) and G-band (1585 cm^−1^), along with a weaker 2D band (2693 cm^−1^). The presence of the D band indicated the existence of defect and partial disorder structures caused by sp^3^ carbon atoms during the oxidation process, while the G band was associated with the in-plane vibration of sp^2^ carbon atoms [22,29]. The intensity ratio of the D-band and G-band (I_D_/I_G_) can be used to evaluate the degree of defectiveness and disorderliness of GO sheets. It is worth noting that the Raman spectra of AGO55, AGO65, AGO75, and AGO85 were without band shifts compared to that of GO. However, the intensity ratio of the D-band to G-band (I_D_/I_G_) increased from 1.43 (GO) to 1.72 (AGO55), 1.89 (AGO65), 1.82 (AGO75), and 1.76 (AGO85), demonstrating that the structure of AGO was more disordered after the grafting of the amino group. AGO65 had the highest I_D_/I_G_, indicating that 65 °C was more suitable for the reaction of GO and 2-aminomalonamide, which was conducive to the grafting of amino groups on the surface of GO. 

Figure 2c shows the FT-IR spectra of GO and AGO. The prominent absorption peaks at 3430, 1725, 1630, and 1100 cm^−1^ were attributed to −OH, C=O, C-OH, and C−O−C functionalities, respectively. After the reaction with 2-aminomalonamide, the intensity of the C=O peak (1725 cm^−1^) decreased, and the C–N peak (1400 cm^−1^) appeared in contrast to the GO spectrum. In addition, the intensity of the C–N peak apparently rose with increasing temperatures. This suggests that the graft modification of GO with amino groups was successfully achieved, and the grafting of amino functional groups can be facilitated by increasing the temperature.

**Figure 3:**
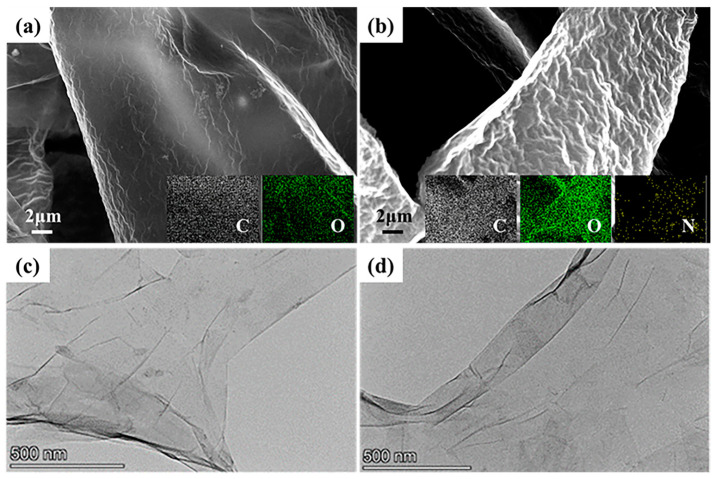
SEM and EDS mapping images of GO (**a**) and AGO65 (**b**), TEM images of GO (**c**) and AGO65 (**d**).

The results indicated that the optimal reaction temperature of GO and 2-aminomalonamide was 65 °C. Consequently, further characterization was conducted on the resulting composites obtained at this temperature. SEM and TEM were employed to observe the microscopic morphology of GO and AGO65. The surface of GO exhibited a relatively smooth appearance with prominent undulating folds in Figure 3a. After the grafting of 2-aminomalonamide from Figure 3b, the surface of the AGO was rougher in comparison to that of GO owing to the heating and ultrasonic treatment in the modification process, and the N element was detected on the surface of AGO. The rise in surface roughness hindered the agglomeration of AGO and facilitated AGO uniform dispersion in the epoxy resin coatings. In addition, the increase in shadow area in the TEM image of AGO indicated an increase in layer thickness (Figure 3c,d). Both the SEM and TEM results indicated that the amino group was successfully grafted onto the surface of GO after 2-aminomalonamide modification. 

**Figure 4:**
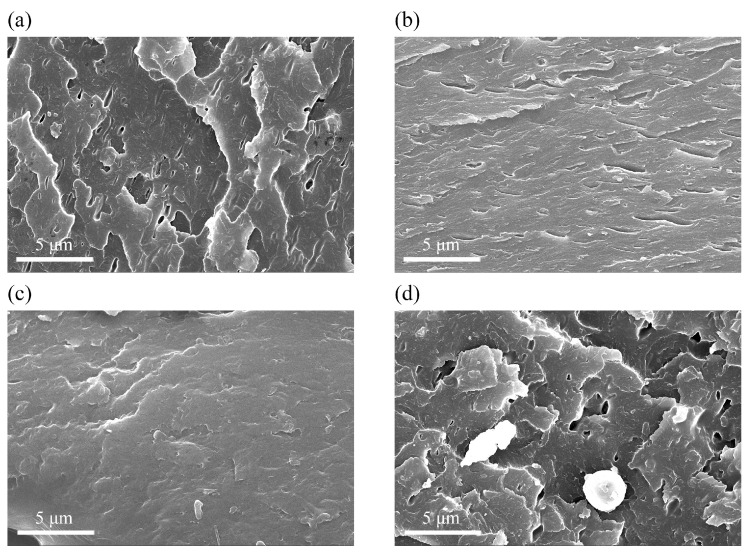
The fracture surfaces for (**a**) WEP, (**b**) AGO05/WEP, (**c**) AGO15/WEP, and (**d**) AGO30/WEP coatings.

The dispersion of different amounts of AGO in the WEP was conducted by analyzing the fracture surfaces. All samples were treated with liquid nitrogen prior to the SEM analysis. As shown in Figure 4a, the fracture surface of the WEP exhibited noticeable pore defects, which were caused by solvent volatilization during the curing process of the waterborne epoxy coatings. In this case, the micropores can serve as potential pathways for corrosive media to infiltrate, leading to the corrosion of the metal substrate [30]. The defects and pores decreased after the incorporation of AGO into the epoxy resins, and the fracture surface of the AGO15/WEP composite coating displayed a denser morphology, suggesting that the addition of 0.15 wt.% of AGO effectively blocked defects and pores within the coating to block the penetration of corrosive mediums. However, the continuous addition of AGO led to the appearance of agglomeration and the uneven distribution of AGO (Figure 4d), which led to the defects and pores in the AGO30/WEP composite coating.

### 3.2. Anti-Corrosive Performance of AGO/WEP Composite Coatings

**Figure 5:**
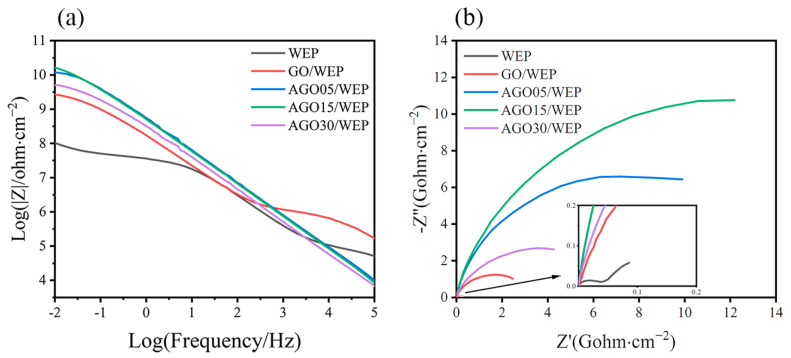
Bode diagrams (**a**) and Nyquist diagrams (**b**) of coatings after immersion in a 3.5 wt.% NaCl solution for 24 h.

EIS was used to investigate the electrochemical behavior and protective performance of the composite coatings. The Nyquist and Bode plots of the coatings after immersion in a 3.5 wt.% NaCl solution for 24 h are shown in Figure 5. Typically, an impedance modulus at 0.01 Hz (|Z|_0.01Hz_) is utilized to evaluate the protective efficacy of coatings, with higher |Z|_0.01Hz_ values signifying enhanced corrosive resistance [31]. The |Z|_0.01Hz_ after 24 h immersion was 1.03 × 10^8^, 2.67 × 10^9^, 1.17 × 10^10^, 1.63 × 10^10^, and 5.01 × 10^9^ ohm·cm^−2^ for the WEP, GO/WEP, AGO05/WEP, AGO15/WEP, and AGO30/WEP coatings, respectively (Figure 5a). The corrosion resistance of the composite coatings was significantly improved after the application of GO and AGO in the WEP. Figure 5b shows that the radius of capacitive arcs first increased and then decreased with an increase in AGO65, and AGO15/WEP with 0.15 wt.% AGO65 had the largest radius, which was consistent with the analysis of the fracture surfaces. In addition, the Nyquist diagram of the GO and AGO coating samples exhibited a larger capacitive loop and only one time constant, suggesting that the corrosive medium permeated the coatings but did not reach the interface between the coating and the metal substrate. The ionic resistance (barrier action) of the coatings controlled the main corrosion process in these samples [32].

**Figure 6:**
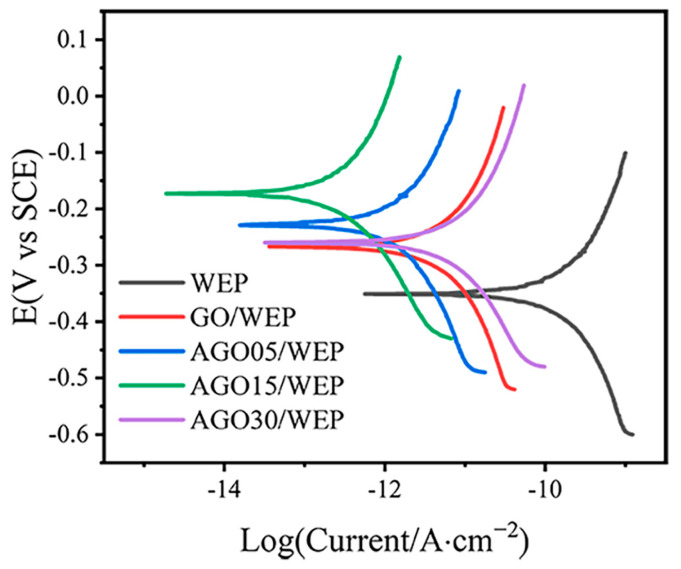
Tafel curves of WEP, GO/WEP, and AGO/WEP coatings.

The Tafel curves of the WEP, GO/WEP, AGO05/WEP, AGO15/WEP, and AGO30/WEP coatings are shown in Figure 6, and the electrochemical parameters are summarized in Table 4. Both the GO/WEP and AGO/WEP composite coatings demonstrated a decrease in i_corr_ compared to the WEP coating, indicating their enhanced corrosion resistance properties. Due to the beneficial effect of incorporating AGO in the shielding and anti-corrosion performance of waterborne epoxy coatings, the AGO15/WEP coating exhibited the lowest corrosion current density (3.11 × 10^−13^ A·cm^−2^). Furthermore, the calculated corrosion rate of AGO15/WEP was 3.63 × 10^−9^ mm/year, which was obviously less than that of WEP (1.79 × 10^−6^ mm/year). 

**Figure 7:**
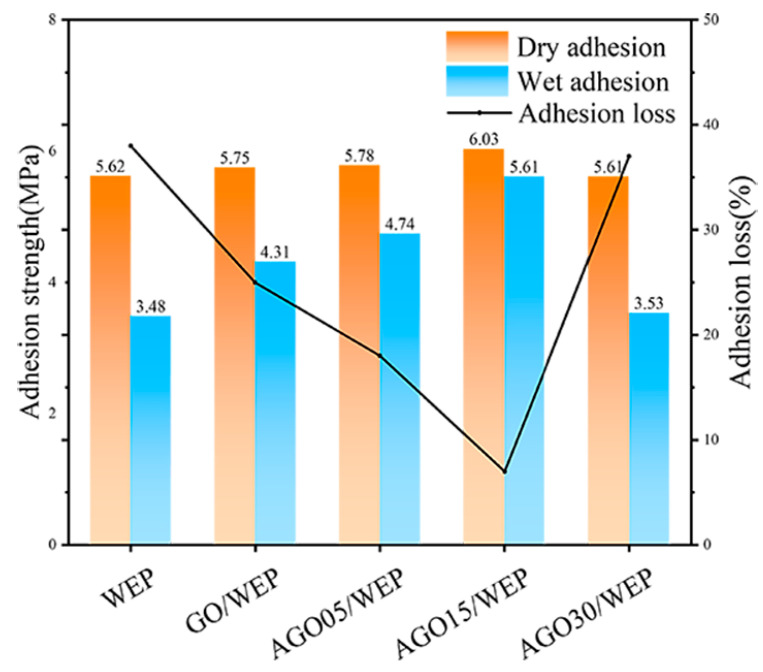
The adhesion of WEP, GO/WEP, and AGO/WEP coatings.

During the process of the metal matrix corrosion, the generation of OH^−^ at the coating–metal interface (2H_2_O + O_2_ + 4e^−^ → 4OH^−^) led to a localized increase in pH at the interface, resulting in enhanced coating hydrolysis and reduced coating adhesion [33]. Figure 7 shows the adhesion strength of the composite coatings. The dry and wet adhesion of the WEP was measured to be 5.62 and 3.48 MPa, respectively. And both the dry and wet adhesion of the AGO15/WEP coating significantly increased, with a dry adhesion of 6.03 MPa and a wet adhesion of 5.61 MPa, respectively. Notably, the adhesion loss of 7% for AGO15/WEP compared to that of 38% for the WEP coating indicated that AGO filled the defects and pores of WEP and improved the interaction force between the coating and metal substrate. In addition, the expansion benefit of the corrosive medium in the coating significantly increased due to the 0.15 wt.% addition of AGO, thus delaying the corrosion process of the steel substrate.

**Figure 8:**
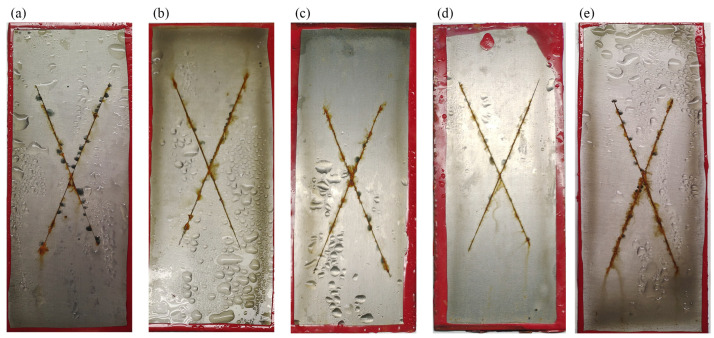
Surface morphology of the (**a**) WEP, (**b**) GO/WEP, (**c**) AGO05/WEP, (**d**) AGO15/WEP, and (**e**) AGO30/WEP coatings after the salt spray test for 200 h.

The salt spray test further evaluated the anti-corrosion performance of the composite coatings. Figure 8a illustrates the presence of significant corrosion products and numerous bubbles surrounding the scratches and beneath the coating, indicating the poor shielding action and corrosion resistance of the WEP. By incorporating 0.15 wt.% of GO into the system, the corrosion products around the scratches were considerably reduced; however, an abundance of bubbles persisted, as shown in Figure 8b. Notably, the corrosion degree of the plate decreased first and then increased with the addition of AGO from 0.05 to 0.30 wt.%. And Figure 8d demonstrates the remarkable absence of corrosion products and bubbles at the surface of AGO15/WEP, which was attributed to the uniform dispersion of AGO throughout the coating, highlighting its effective shielding action and corrosion resistance. However, the addition of excess AGO into AGO30/WEP resulted in a gradual increase in corrosion products and bubbles, primarily due to the uneven distribution of AGO.

### 3.3. Corrosion Protective Mechanism

**Figure 9:**
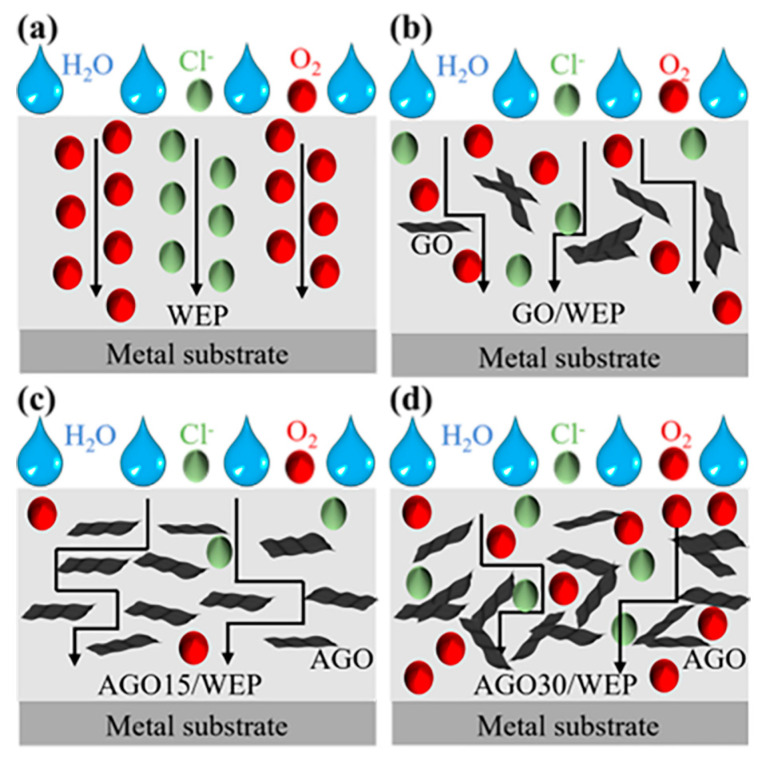
Schematic diagram of the corrosion protection mechanism for (**a**) WEP, (**b**) GO/WEP, (**c**) AGO15/WEP, and (**d**) AGO30/WEP coatings.

The corrosion protection mechanisms of WEP, GO/WEP, AGO15/WEP, and AGO30/WEP composite coatings are proposed in Figure 9. The redox reaction caused by the corrosive mediums (H_2_O, O_2_, and Cl^−^) was the main cause of the corrosion and degradation of the metal substrate. These corrosive media can penetrate the coating through cracks and pores, eventually reaching the interface between the coating and the metal substrate [34]. Due to the limited barrier capacity of the WEP coating, corrosive media quickly reached the surface of the metal substrate and caused the metal corrosion (Figure 9a). The corrosion abated with the introduction of GO into the WEP. However, the corrosion protection of the GO/WEP remained underutilized due to the inevitable aggregation of GO (Figure 9b). The AGO15/WEP composite coating yielded optimum corrosion protection performance due to two main reasons: one is that AGO filled the defects and pores in the waterborne epoxy coatings to block the penetration of corrosive media; the other was that the modification of 2-aminomalonamide enhanced the dispersibility of AGO and improved the “maze effect” of the coating, thus delaying the corrosion process of the underlying carbon steel substrate (Figure 9c). It is worth noting that excessive AGO addition led to a reduction in the corrosion-protective properties, because AGO aggregation within the coatings resulted in increased defects and pores in the coating (Figure 9d). 

## 4. Conclusions

In conclusion, an efficacious method is provided to enhance the anti-corrosion performance of WEP by using the economical industrial byproduct kish graphite as a raw material. The addition of 0.15 wt.% of AGO into WEP resulted in a significant improvement in corrosion protection. After immersion in a 3.5 wt.% NaCl solution, the |Z|_0.01Hz_ was 1.63 × 10^10^ ohm·cm^−2^, which was about two orders higher than that of the WEP (1.03 × 10^8^ ohm·cm^−2^), and the adhesion loss decreased from 38% to 7%. The surface of AGO15/WEP exhibited minimal corrosion products or bubbles after 200 h of salt spray tests. The mechanism of corrosion resistance revealed that AGO could effectively fill micropores owing to its superior dispersibility, preventing the penetration of corrosive media, thus ultimately enhancing corrosion resistance. This research could provide a novel approach for the KFG and graphene composites in anti-corrosion applications. KFG was used as a raw material to prepare GO for improving the anti-corrosion performance of the coating, which has a market application prospect in the corrosion protection of steel. In following research, we will further evaluate the influence of long cycles and environmental factors (temperature, humidity, salt concentration, etc.) on the corrosion resistance of the material.

## Figures and Tables

**Table 1:** The preparation method and application of amino-modified GO.

GO	Modifier	Preparation Method	Application
Angxing Technology Co., Ltd. (Changzhou, China)	Polydopamine	24 h at room temperature	Anti-corrosion coatings [16]
Modified hummers	Cysteine	2 h at room temperature	Aiagnostic agent in nuclear medicine [17]
Modified hummers	(3-aminopropyl) triethoxysilane (APTES)	24 h at 30 °C	Adsorbent for removal of Hg^2+^ and Pb^2+^ from wastewaters [18]
Modified hummers	2-Aminopyridine	24 h at 90 °C with carboxylic acid activators (DCC/DMAP)	Photocatalyst of Water Splitting [19]

**Table 2:** Chemical composition of Q235 steel (mass percentage).

Element	Fe	Mn	Si	P	S	C
wt.%	Balance	0.47	0.28	0.033	0.047	0.17

**Table 3:** Proximate analysis of the purified kish graphite and chemical composition analysis of the ash.

Proximate Analysis		XRF Analysis (wt.%)									
FC (wt.%)	PS (μm)	SiO_2_	Fe_2_O_3_	CaO	Al_2_O_3_	Cr_2_O_3_	ZnO	TiO_2_	MnO	Others	Total
98.47	50–75	56.97	19.89	4.88	3.52	3.07	2.90	2.81	2.26	3.70	100

FC: carbon content; PS: particle size; Others: K_2_O, NiO, MgO, Na_2_O, V_2_O_5_, and P_2_O_5_.

**Table 4:** Electrochemical parameters for WEP, GO/WEP, and AGO/WEP coatings.

Sample	*i_corr_* (A·cm^−2^)	*v_corr_* (mm·Year^−1^)	Rp (Ohm·cm^−2^)
WEP	1.53 × 10^−10^	1.79 × 10^−6^	2.94 × 10^9^
GO/WEP	5.29 × 10^−12^	6.17 × 10^−8^	8.35 × 10^10^
AGO05/WEP	1.39 × 10^−12^	1.64 × 10^−8^	8.60 × 10^11^
AGO15/WEP	3.11 × 10^−13^	3.63 × 10^−9^	1.29 × 10^12^
AGO30/WEP	7.32 × 10^−12^	8.56 × 10^−8^	1.47 × 10^11^

## Data Availability

Data are contained within the article.
